# Effect of pirfenidone on plasma markers of collagen turnover in patients with heart failure, preserved left ventricular ejection fraction and myocardial fibrosis

**DOI:** 10.1136/openhrt-2025-003596

**Published:** 2026-02-10

**Authors:** Nicholas Black, Gavin Lewis, Fardad Soltani, Susanna Dodd, Erik B Schelbert, Susana Ravassa, Begoña López, Arantxa González, John G Cleland, Christopher A Miller

**Affiliations:** 1 BHF Manchester Centre for Heart and Lung Magnetic Resonance Research, Manchester University NHS Foundation Trust, Southmoor Road, Wythenshawe, Manchester, M13 9LT, UK, Manchester, UK; 2 Division of Cardiovascular Sciences, School of Medical Sciences, Faculty of Biology, Medicine and Health, Manchester Academic Health Science Centre, The University of Manchester, Manchester, UK; 3 Liverpool University Hospitals NHS Foundation Trust, Liverpool, UK; 4 Department of Health Data Science, Institute of Population Health, Faculty of Health and Life Sciences, University of Liverpool, Liverpool, UK; 5 Allina Health Minneapolis Heart Institute, Allina Health, Abbott Northwestern Hospital, Minneapolis, Minnesota, USA; 6 Program of Cardiovascular Research, Centro de Investigación Médica Aplicada, Universidad de Navarra, and IdiSNA and CIBERCV, Pamplona, Spain; 7 Department of Cardiology and Cardiac Surgery, Clínica Universidad de Navarra, Pamplona, Spain; 8 British Heart Foundation Centre of Research Excellence, School of Cardiovascular and Metabolic Health, University of Glasgow, Glasgow, UK

**Keywords:** MYOCARDIAL FIBROSIS, Heart Failure, Diastolic, Magnetic Resonance Imaging

## Abstract

**Background:**

Plasma concentrations of procollagen type-I C-terminal pro-peptide (PICP) and collagen type-I C-terminal telopeptide (CITP) may reflect collagen turnover and systemic fibrosis. We investigated the effect of pirfenidone, an anti-fibrotic agent, on PICP and CITP, and their association with myocardial fibrosis, using cardiovascular magnetic resonance to measure extracellular volume (ECV).

**Methods:**

In the trial (Pirfenidone in Patients with Heart Failure and Preserved Left Ventricular Ejection Fraction), PICP, CITP and PICP:CITP ratio were measured at baseline and follow-up in patients with ECV≥27% randomised (n=94) to pirfenidone or placebo, and at baseline only in patients who were not randomised because of ECV<27% (n=13).

**Results:**

There was no association between baseline myocardial ECV and baseline log PICP, log CITP and log PICP:CITP ratio (p=0.19, p=0.13, p=0.60, respectively). Treatment with pirfenidone did not alter PICP, but reduced CITP and increased PICP:CITP ratio at 13 and 26 weeks (all p<0.05) but not at 52 weeks. After multivariable adjustment, there was a weak relationship between change in myocardial ECV and change in log PICP (R^2^ 0.16, p=0.01) and log CITP (R^2^ 0.12, p=0.04), but not log PICP:CITP ratio (p=0.56).

**Conclusions:**

In patients with stable heart failure with preserved ejection fraction, pirfenidone treatment had no sustained effect on plasma levels of PICP and CITP at 52 weeks. Changes in ECV during treatment with pirfenidone are associated with changes in plasma PICP and CITP, suggesting a weak association between changes in collagen volume/mass and plasma markers of collagen turnover.

**WHAT IS ALREADY KNOWN ON THIS TOPIC**

- Procollagen type-I C-terminal pro-peptide (PICP) and collagen type-I C-terminal telopeptide (CITP) have been proposed as plasma biomarkers of collagen turnover and myocardial fibrosis. However, the effect of the anti-fibrotic drug pirfenidone on plasma levels of PICP and CITP, and the correlation with changes in myocardial fibrosis, as measured by cardiovascular magnetic resonance (CMR) extracellular volume (ECV), has not been described.

**WHAT THIS STUDY ADDS**

- This study demonstrates that in patient with heart failure with preserved ejection fraction and more severe myocardial fibrosis (ECV ≥27%), pirfenidone treatment had no sustained effect on plasma levels of PICP and CITP at 52 weeks (although an increase in PICP:CITP ratio was seen at 13 and 26 weeks). After multivariable adjustment, significant although weak associations were noted between change in PICP and CITP and change in myocardial ECV with pirfenidone treatment.

**HOW THIS STUDY MIGHT AFFECT RESEARCH, PRACTICE OR POLICY**

- This study highlights both the potential and limitations of PICP and CITP as plasma biomarkers of collagen turnover. They respond only transiently to systemic anti-fibrotic therapy with pirfenidone and correlate weakly with changes in myocardial fibrosis, as assessed by CMR ECV. These biomarkers may reflect changes in the rate of turnover of collagen rather than reflecting the mass of collagen per se.

## Introduction

 Myocardial fibrosis is a key pathophysiological mechanism implicated in many cardiovascular diseases.^1^ Cardiovascular magnetic resonance (CMR) extracellular volume (ECV) represents the most validated non-invasive measure of myocardial fibrosis^2^ and is consistently predictive of adverse outcome across all cardiovascular conditions in which it has been investigated.^3^,^9^ Myocardial fibrosis is widely recognised to be an important therapeutic target, and there is growing interest in the use of CMR ECV to identify people for inclusion in trials of anti-fibrotic therapy.^10 11^ However, CMR is a relatively uncommon and costly imaging modality.

Plasma biomarkers have been proposed as a cheaper and more accessible alternative for the non-invasive assessment of myocardial fibrosis and collagen metabolism. While many plasma biomarkers of myocardial fibrosis have been proposed, few have been shown to correlate with histologically assessed myocardial fibrosis. Two collagen type I-derived peptides, procollagen type-I C-terminal pro-peptide (PICP) and collagen type-I C-terminal telopeptide (CITP), have been shown to correlate with histological fibrosis on endomyocardial biopsy,^12^ although the relationship is variable.

Pirfenidone, an oral agent licensed for the treatment of idiopathic pulmonary fibrosis, mediates anti-fibrotic effects by inhibiting transforming growth factor-beta (TGF-ß) and fibroblast proliferation and differentiation.^13^ In the PIROUETTE trial (Pirfenidone in Patients with Heart Failure and Preserved Left Ventricular Ejection Fraction) of patients with heart failure, preserved ejection fraction (HFpEF) and a high ECV (≥27%), pirfenidone reduced myocardial fibrosis, as assessed by ECV on CMR.^14^

This trial provides an opportunity to investigate the relationship between plasma markers of collagen turnover and ECV, and the effects of pirfenidone. 

## Methods

### Trial design

In the PIROUETTE trial (ClinicalTrials.gov NCT02932566), 94 patients with HFpEF and myocardial fibrosis were randomised to pirfenidone or placebo for 52 weeks.^14 15^ Patients were recruited between 7 March 2017 and 19 December 2018. Eligibility requirements included age ≥40 years, symptoms and signs of heart failure (HF), left ventricular ejection fraction ≥45% and elevated plasma concentrations of natriuretic peptides (brain natriuretic peptide ≥100 pg/mL or N‐terminal pro‐B‐type natriuretic peptide (NT‐proBNP) ≥300 pg/mL; or brain natriuretic peptide ≥300 pg/mL or NT‐proBNP≥900 pg/mL if in atrial fibrillation). Eligible patients underwent CMR and those with evidence of myocardial fibrosis, defined as an ECV of ≥27%, were randomised in a 1:1 ratio to treatment with either pirfenidone 2403 mg daily or matching placebo for 52 weeks using block randomisation, stratified by sex. Patients with ECV <27% were entered into a registry (n=13). Key exclusion criteria included alternative causes of patients’ symptoms such as pulmonary disease, anaemia or obesity; pericardial constriction, hypertrophic cardiomyopathy or infiltrative cardiomyopathy; and contraindications to MRI. The primary outcome was change in myocardial fibrosis, measured using CMR ECV, from baseline to 52 weeks.

The trial was sponsored by Manchester University NHS Foundation Trust and Liverpool Clinical Trials Centre, a UK Clinical Research Collaboration Registered Clinical Trials Unit, was the clinical trials unit. The investigational medicinal product was gifted by Roche Products. Roche Products had no role in study design or data analysis. The study protocol was approved by a research ethics committee and trial conduct was overseen by a trial steering committee. Patients were identified at six hospitals in the UK. Study visits took place at Manchester University National Health Service (NHS) Foundation Trust. All patients provided written informed consent.

### Fibrosis biomarker analysis

Patients provided written informed consent for blood samples to be stored in a central biorepository at baseline and at 13, 26 and 52 weeks postrandomisation. At baseline, plasma samples were available for 93 (of 94) randomised patients and 13 (of 13) registry patients. During follow-up, plasma samples were available for 83, 61 and 80 randomised patients at 13, 26 and 52 weeks, respectively. CITP was measured by radio-immunoassay (UniQ Aidian) and PICP by enzyme immune-assay (METRA; Quidel Corporation). All interassay and intra-assay variations were below 10%.

### Statistical analysis

The original trial design included prospective sample storage at baseline 13, 26 and 52 weeks for unspecified future analyses. Fibrosis biomarkers were not included in the original Statistical Analysis Plan and thus the analyses now reported are considered post hoc. The trial was not powered for secondary outcomes; thus, the findings of this study are exploratory.

Continuous data are presented as median±IQR. Categorical data are presented as counts and percentages. The distribution of PICP, CITP and PICP:CITP ratio data was non‐normal (online supplemental figure1, Shapiro-Wilk tests for normality were p<0.001). Data underwent natural logarithm transformation to normalise (online supplemental figure 2).

Treatment‐related analyses were conducted on an intention‐to‐treat basis. Log PICP, log CITP and log PICP:CITP ratio at week 52 were compared between treatment groups using analysis of covariance (ANCOVA), adjusting for baseline concentrations, stratification factor (sex), estimated glomerular filtration rate (eGFR) at 52 weeks and treatment group. Power analysis (G*Power) demonstrated that 94 randomised patients achieved a statistical power of 67% to predict a medium effect size (Cohen f 0.25) with a type 1 error rate of 0.05.^16^ The impact of pirfenidone treatment on log PICP, log CITP and log PICP:CITP ratio over time was also determined using a repeated-measure linear mixed model with an unstructured covariance structure. Time (months), baseline concentrations, sex, eGFR at 52 weeks, treatment group and interaction between time and treatment group were included as fixed effects. Biomarker concentrations at different time points were compared using a two-sample t-test.

Univariable and multivariable regression models were used to assess the relationships between change in myocardial ECV from baseline to week 52, and change in log PICP, log CITP and log PICP:CITP ratio. Variables for which p values were <0.3 in the univariable analyses proceeded to combined forward and reverse stepwise Akaike information criterion (AIC) selection. The chosen variables were then included in multivariable regression models, alongside log PICP, log CITP or log PICP:CITP ratio. The same methods were also used to assess the relationship between baseline myocardial ECV and baseline log PICP, log CITP and log PICP:CITP ratio. Baseline biomarker concentrations between randomised (ECV≥27%) and registry (ECV<27%) were compared using a two-sample t-test.

## Results

### Patient characteristics

Baseline characteristics are summarised in table 1. At the end of the trial, 12 of the 94 patients who were randomised had withdrawn from the study and 2 had died. No patient was lost to follow‐up. Therefore, data from 94 randomised patients at baseline and 80 patients at 52 weeks were included in the analysis.

**Table 1:** Table of baseline characteristics

	Randomised (ECV≥27%)		Registry (ECV<27%, n=13)
Demographics	Placebo (n=47)	Pirfenidone (n=47)	
Age (years)	81 (76–83)	78 (72–82)	74 (72–76)
Female sex (%)	21 (44.7%)	22 (46.8%)	9 (69.2%)
White ethnicity (%)	43 (91.5%)	45 (95.7%)	13 (100%)
BMI (kg/m^2^)	29 (26–33)	31 (27–34)	33 (31–35)
NYHA class (%):			
I	5 (10.6%)	0 (0.0%)	1 (7.7%)
II	19 (40.4%)	26 (55.3%)	11 (84.6%)
III	23 (48.9%)	21 (44.7%)	1 (7.7%)
IV	0 (0.0%)	0 (0.0%)	0 (0.0%)
Comorbidity			
Hypertension (%)	40 (85.1%)	39 (83.0%)	10 (76.9%)
Diabetes (%)	12 (25.5%)	16 (34.0%)	2 (15.4%)
Atrial fibrillation (%)	27 (57.4%)	27 (57.4%)	4 (30.8%)
Stroke (%)	5 (10.6%)	5 (10.6%)	1 (7.7%)
Hyperlipidaemia (%)	12 (25.5%)	10 (21.3%)	1 (7.7%)
Ischaemic heart disease (%)	19 (40.4%)	17 (36.2%)	4 (30.8%)
Prior HF hospitalisation (%)	7 (14.9%)	8 (17.0%)	0 (0.0%)
COPD (%)	7 (14.9%)	5 (10.6%)	0 (0.0%)
Current smoker (%)	0 (0.0%)	1 (2.13%)	0 (0.0%)
Ex-smoker (%)	17 (36.2%)	15 (31.9%)	8 (61.5%)
Laboratory measurements			
Haemoglobin (g/L)	127 (117–136)	132 (122–142)	133 (130–137)
White cell count (10^9^/L)	7.4 (6.3–9.0)	7.4 (6.8–8.7)	6.8 (6.2–7.8)
Sodium (mmol/L)	139 (136–140)	139 (137–141)	140 (138–142)
Creatinine (umol/L)	109 (80–131)	94 (81–107)	93 (82–111)
eGFR (mL/min/1.73 m^2^)	53 (38–65)	58 (46–76)	52 (42–74)
HsTropT (pg/mL)	26 (15–38)	17 (11–25)	13 (10–17)
NTproBNP (pg/mL)	1372 (626–2817)	975 (445–2064)	423 (324–803)
GDF-15 (pg/mL)	3046 (1970–5422)	2388 (1749–3116)	1724 (1563–1954)
PICP (ng/mL)	147.0 (115.0–207.0)	142.0 (117.0–200.0)	125.6 (120.3–161.9)
log PICP	5.0 (4.8–5.3)	5.0 (4.8–5.3)	4.9 (4.8–5.1)
CITP (ng/mL)	6.9 (4.6–9.4)	5.2 (4.3–7.6)	4.8 (3.7–5.6)
log CITP	1.9 (1.5–2.2)	1.6 (1.5–2.0)	1.6 (1.3–1.7)
PICP:CITP ratio	24.7 (18.9–30.6)	27.6 (19.1–35.4)	28.2 (26.2–34.4)
log PICP:CITP ratio	3.2 (2.9–3.4)	3.3 (3.0–3.6)	3.3 (3.3–3.5)
Cardiovascular MRI			
LVMassi (g/m^2^)	66 (53–73)	62 (54–71)	66 (61–73)
LVEDVi (mL/m^2^)	60 (51–75)	59 (50–74)	66 (63–74)
LVESVi (mL/m^2^)	21 (15–31)	19 (14–26)	20 (18–23)
LVEF (%)	65 (55–69)	67 (60–70)	71 (68–75)
RVEDVi (mL/m^2^)	67 (57–77)	68 (60–79)	72 (65–77)
RVESVi (mL/m^2^)	34 (28–42)	31 (26–39)	29 (25–36)
RVEF (%)	51 (43–57)	53 (48–59)	58 (56–65)
LAVi (mL/m^2^)	69 (58–85)	68 (56–83)	59 (56–71)
RAVi (mL/m^2^)	65 (49–89)	65 (55–81)	57 (54–66)
Aortic distensibility (10^–3^/mm Hg)	1.4 (1.0–1.9)	1.4 (1.0–2.1)	0.9 (0.6–1.5)
Infarct LGE (%)	12 (25.5%)	8 (17%)	0 (0.0%)
Non-ischaemic LGE (%)	18 (38.3%)	12 (25.5%)	1 (7.7%)
Myocardial ECV (%)	30.4 (28.3–32.2)	28.9 (27.6–31.0)	24.7 (24.5–24.9)

Values are median ±(IQR).

BMI, body mass index; CITP, collagen type-I C-terminal telopeptide; COPD, chronic obstructive pulmonary disease; ECV, extracellular volume; eGFR, estimated glomerular filtration rate; GDF-15, growth differentiation factor 15; HF, heart failure; hsTropT, high sensitivity troponin T; LAVI, indexed left atrial volume; LGE, late gadolinium enhancement; LVEDVi, indexed left ventricle end-diastolic volume; LVEF, left ventricle ejection fraction; LVESVi, indexed left ventricle end-systolic volume; LVMassi, indexed left ventricle mass; NTproBNP, N-terminal pro B-type natriuretic peptide; NYHA, New York Heart Failure Association; PICP, procollagen type-I C-terminal pro-peptide; RAVI, indexed right atrial volume; RVEDVi, indexed right ventricle end diastolic volume; RVEF, right ventricle ejection fraction; RVESVi, indexed right ventricle end systolic volume.

### Effect of pirfenidone on plasma biomarkers of fibrosis

**Figure 1:**
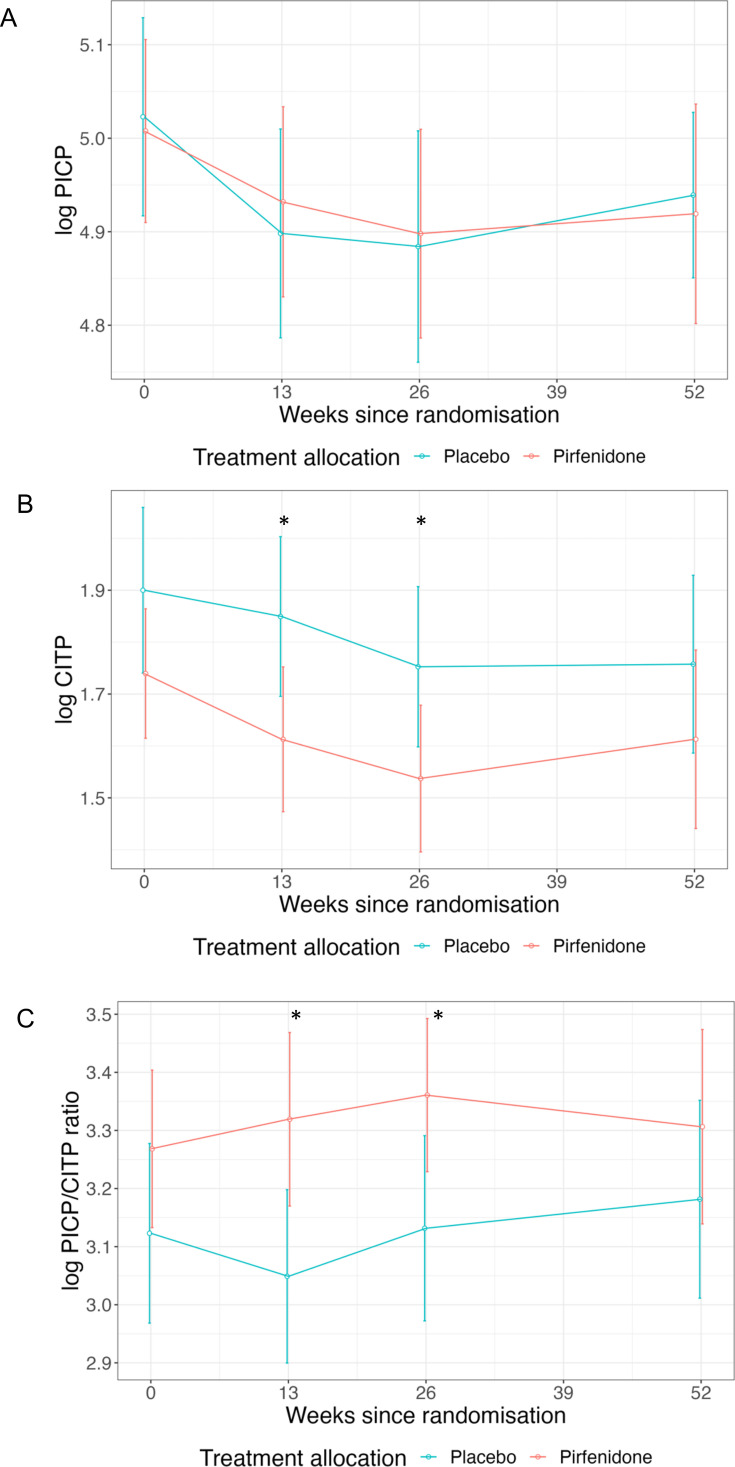
Concentrations of (**A**) log PICP, (**B**) log CITP and (**C**) log PICP:CITP ratio from baseline to 52 weeks in patients randomised to pirfenidone and placebo. Values are mean±95% CIs. *p<0.05 for two sample t-test. CITP, collagen type-I C-terminal telopeptide; PICP, procollagen type-I C-terminal pro-peptide.

Concentrations of log PICP, log CITP and log PICP:CITP ratio in patients randomised to pirfenidone or placebo are shown in figure 1 and table 2. Treatment with pirfenidone did not alter week 52 log PICP, log CITP or log PICP:CITP ratio (ANCOVA p=0.91, p=0.78 and p=0.88, respectively). However, pirfenidone treatment reduced log CITP and increased log PICP:CITP ratio at 13 and 26 weeks (p<0.05, figure 1 and table 2). Repeated measures linear mixed modelling also suggested a trend towards an increase in log PICP:CITP ratio with pirfenidone treatment (p=0.008 for treatment effect, and p=0.064 for interaction between time and treatment effect, online supplemental table 1).

**Table 2:** Concentrations of PICP, CITP and PICP:CITP ratio from baseline to 52 weeks in patients randomised to pirfenidone and placebo

	Week 0			Week 13			Week 26			Week 52		
	Placebo	Pirfenidone	P value	Placebo	Pirfenidone	P value	Placebo	Pirfenidone	P value	Placebo	Pirfenidone	P value
Number tested	47	46	–	44	39	–	34	27	–	41	39	–
Log PICP	5.0 (0.4)	5.0 (0.3)	0.84	4.9 (0.4)	4.9 (0.3)	0.66	4.9 (0.4)	4.9 (0.3)	0.87	4.9 (0.3)	4.9 (0.4)	0.79
Log CITP	1.9 (0.6)	1.7 (0.4)	0.12	1.8 (0.5)	1.6 (0.4)	**0.03**	1.8 (0.5)	1.5 (0.4)	**0.05**	1.8 (0.6)	1.6 (0.5)	0.25
Log PICP:CITP ratio	3.1 (0.5)	3.3 (0.5)	0.17	3.0 (0.5)	3.3 (0.5)	**0.01**	3.1 (0.5)	3.4 (0.3)	**0.03**	3.2 (0.6)	3.3 (0.5)	0.31

Values are mean±(SD).

P values for two sample t-test.

Significant P values <0.05 in bold

CITP, collagen type-I C-terminal telopeptide; PICP, procollagen type-I C-terminal pro-peptide.

### Association between change in myocardial ECV and change in circulating biomarkers of fibrosis

**Figure 2:**
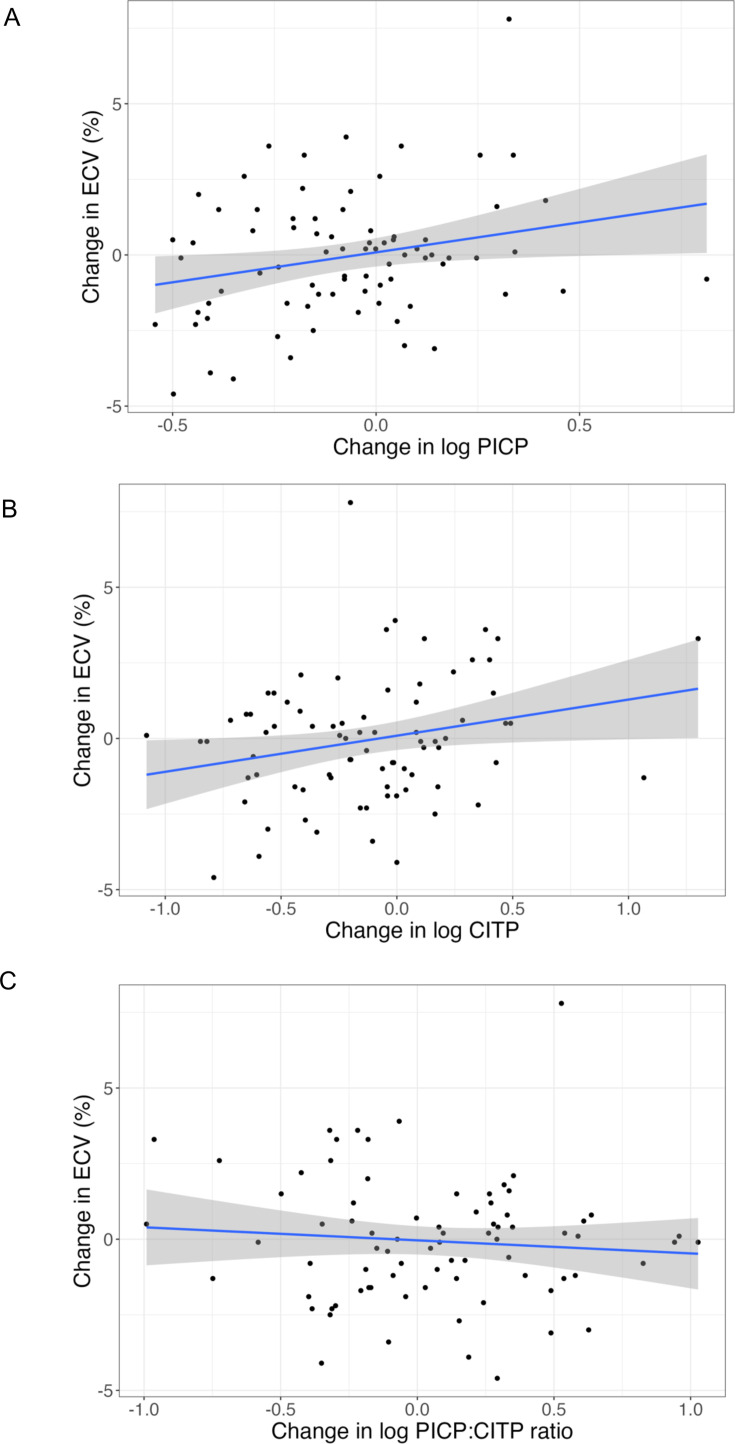
Univariable associations between change in ECV and change in (**A**) log PICP, (**B**) log CITP and (**C**) log PICP:CITP ratio, from baseline to week 52. Regression line (blue) and 95% CI (grey). CITP, collagen type-I C-terminal telopeptide; ECV, extracellular volume; PICP, procollagen type-I C-terminal pro-peptide.

Weak univariate associations were noted between an increase in myocardial ECV from baseline to week 52 and increase in log PICP (R^2^ 0.05, p=0.03) and log CITP (R^2^ 0.05, p=0.03), but not log PICP:CITP ratio (p=0.44) (figure 2 and online supplemental table 2). After multivariable adjustment, independent determinants of change in myocardial ECV included atrial fibrillation, diabetes, ethnicity and white cell count (online supplemental tables 3–5). After multivariable adjustment, there was a significant association between change in myocardial ECV and log PICP (R^2^ 0.16, p=0.01) and log CITP (R^2^ 0.12, p=0.04), but not log PICP:CITP ratio (p=0.56) (table 3).

**Table 3:** Multivariable adjusted associations between change in myocardial ECV and change in circulating biomarkers from baseline to week 52

Variable	Multivariable associations				
	Regression coefficient (SE)	95% CI	T statistic	P value	Adjusted R^2^
Change in log PICP*	0.57 (0.22)	0.14 to 1.00	2.63	**0.01**	0.16
Change in log CITP*	0.46 (0.22)	0.01 to 0.90	2.05	**0.04**	0.12
Change in log PICP:CITP ratio*	−0.13 (0.23)	−0.59 to 0.32	−0.58	0.56	0.07

Change in log PICP multivariable model adjusted for atrial fibrillation, diabetes, indexed left ventricular mass, ethnicity and white cell count. Change in log CITP and log PICP:CITP ratio multivariable models adjusted for atrial fibrillation, indexed left ventricular mass and white cell count.

Significant P values <0.05 in bold

* Regression coefficients standardised to 1 SD change in continuous variables.

CITP, collagen type-1 C-terminal telopeptide; ECV, extracellular volume; PICP, procollagen type-I C-terminal pro-peptide;

### Association between baseline myocardial ECV and circulating biomarkers of fibrosis

**Figure 3:**
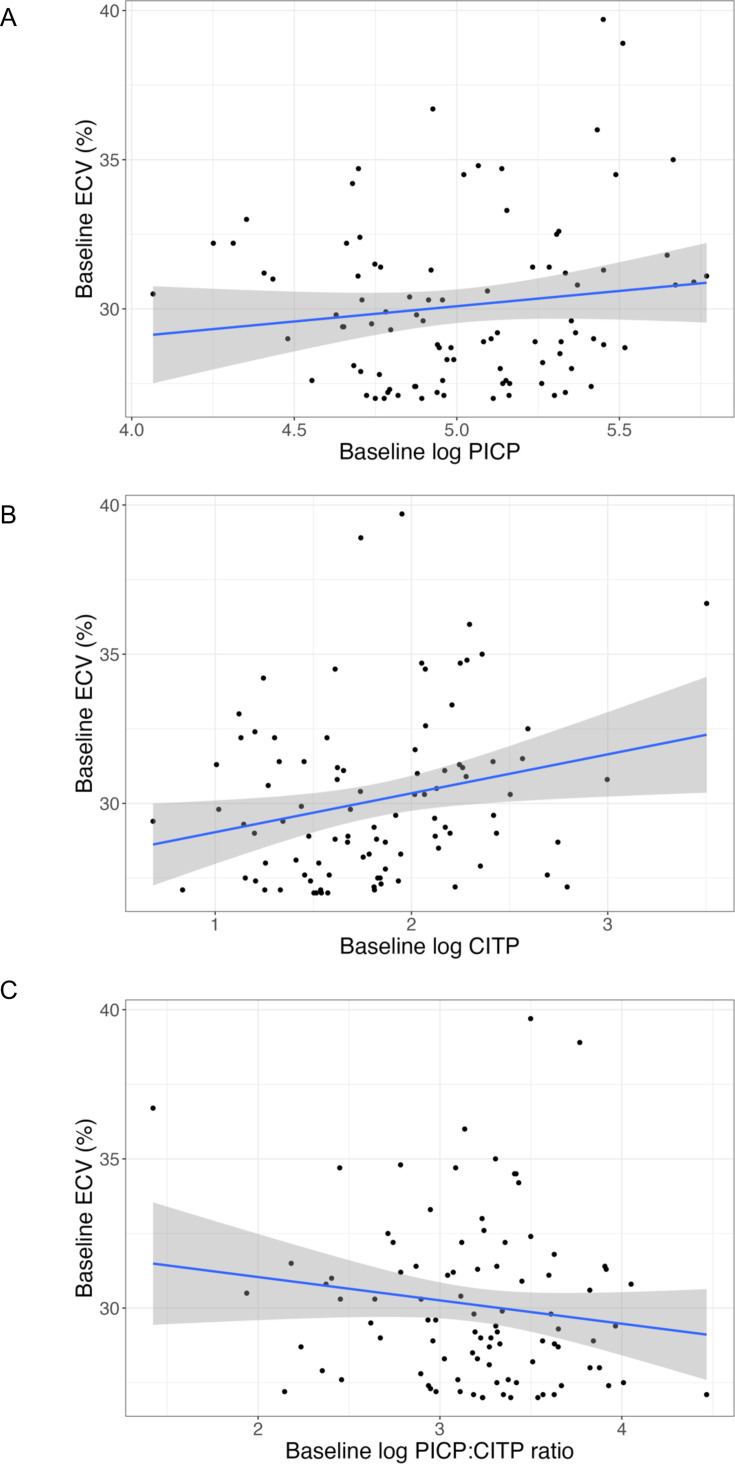
Univariable associations between baseline ECV and baseline (**A**) log PICP, (**B**) log CITP and (**C**) log PICP:CITP ratio. Regression line (blue) and 95% CI (grey). CITP, collagen type-I C-terminal telopeptide; ECV, extracellular volume; PICP, procollagen type-I C-terminal pro-peptide.

At baseline, a weak univariate association was noted between myocardial ECV and plasma concentrations of log CITP (R^2^ 0.05, p=0.02), but not log PICP (p=0.21) or log PICP:CITP ratio (p=0.17) (figure 3 and online supplemental table 6). After multivariable adjustment, independent determinants of baseline myocardial ECV included atrial fibrillation, haemoglobin, hyperlipidaemia, indexed left ventricular mass, baseline log NT-proBNP and stroke (online supplemental tables 7–9). After multivariable adjustment, there was no association between baseline myocardial ECV and baseline concentrations of log PICP, log CITP and log PICP:CITP ratio (p=0.19, p=0.13, p=0.60, respectively, table 4).

**Table 4:** Multivariable adjusted associations between baseline myocardial ECV and baseline circulating biomarkers

Variable	Multivariable associations				
	Regression coefficient (SE)	95% CI	T statistic	P value	Adjusted R^2^
Log PICP*	0.33 (0.25)	−0.17 to 0.84	1.31	0.19	0.29
Log CITP*	0.41 (0.27)	−0.12 to 0.95	1.53	0.13	0.30
Log PICP:CITP ratio*	−0.15 (0.27)	−0.69 to 0.40	−0.53	0.60	0.28

Multivariable models adjusted for atrial fibrillation, body mass index, haemoglobin, hyperlipidaemia, indexed left ventricular mass, baseline log NT-proBNP, and stroke.

* Regression coefficients standardised to 1 SD change in continuous variables.

CITP, collagen type-1 C-terminal telopeptide; ECV, extracellular volume; NT-proBNP, N-terminal pro B-type natriuretic peptide; PICP, procollagen type-I C-terminal pro-peptide;

Baseline concentrations of CITP were higher in patients with ECV≥27% compared with ECV<27% (p=0.02, online supplemental table 10). There were no differences in concentrations of PICP and PICP:CITP ratio (p=0.32 and p=0.11, respectively, online supplemental table 10).

## Discussion

For patients with HFpEF and more severe myocardial fibrosis (ECV≥27%), pirfenidone reduced myocardial fibrosis over 52 weeks but may have had only a modest and transient effect on plasma biomarkers of systemic collagen turnover. This is perhaps not surprising. Changes in plasma collagen biomarkers may reflect changes in the rate of turnover of collagen rather than reflecting the mass of collagen. If so, the largest effect of pirfenidone on plasma biomarkers might appear shortly after initiation and then fade as a new steady state is achieved. In contrast, there is likely to be little change in the myocardial collagen volume/mass in the first few weeks after initiating pirfenidone, with differences appearing only after several months. Differences in the time course of changes in collagen turnover and change in volume/mass may account for the weak relationship between changes in myocardial ECV and plasma biomarkers. Moreover, plasma biomarkers reflect systemic collagen metabolism, but only a very small amount of total body collagen is in the heart. Assessment of fibrosis in specific organs and assessment of systemic collagen metabolism may be considered complementary approaches to the assessment of fibrosis and of anti-fibrotic therapies. However, the trend towards an increase in PICP:CITP ratio suggests a shift towards decreased collagen degradation, and hence the opposite of what might be expected from systemic anti-fibrotic therapy.

While several plasma biomarkers of fibrosis mass or activity have been proposed (eg, galectin-3, growth differentiation factor-1), collagen-derived peptides are the most validated.^17^ PICP is formed from the enzymatic cleavage of procollagen type I into collagen type I and represents a marker of collagen synthesis.^18^ In contrast, CITP is formed from the enzymatic degradation of collagen type I and represents a marker of collagen degradation.^18^ The PICP:CITP ratio is therefore hypothesised to be a dynamic marker of collagen turnover, with higher ratios representing predominant collagen synthesis and lower ratios representing predominant collagen degradation.

In reality, plasma biomarker concentrations are determined by collagen turnover in many different tissue compartments, predominantly extra-cardiac in origin, for example, bone. Even within the heart, the relationship between collagen peptides and fibrosis burden is complicated by the dynamics of collagen metabolism (transient vs steady-state change), broad type of fibrosis (replacement vs interstitial), and mechanism of injury (ischaemic vs non-ischaemic).^18 19^ As a result, the relationship between collagen biomarkers and myocardial fibrosis burden is variable. Markers of collagen synthesis, PICP and procollagen type-III N-terminal pro-peptide (PIIINP), consistently show a positive correlation with the histologically assessed collagen volume fraction (CVF), although the association is variable (R^2^ 0.22–0.77).^20^,^22^ The association between CITP and CVF is inconsistent, with studies reporting both positive^20^ and negative associations.^23^

Several studies have investigated the effects of renin-angiotensin-aldosterone (RAAS) inhibitors on plasma collagen biomarkers. A shift towards net collagen degradation would be expected, with a corresponding reduction in markers of collagen synthesis (PICP/PIIINP) and an increase in markers of collagen breakdown (CITP), at least in the short term. In patients with HFpEF, treatment with sacubitril valsartan was associated with increased concentrations of CITP at 16 weeks, but not at 48 weeks.^24^ In the HOMAGE trial of patients with, or at high risk of, coronary disease and raised NT-proBNP, spironolactone was associated with reductions in the concentration of PICP and in the PICP:CITP ratio, with effects appearing within 1 month and persisting for the following 9 months.^25^ Similarly, spironolactone was associated with a significant reduction in PICP in a pooled analysis of patients with stage B HF (HOMAGE) and HFpEF (Treatment of Preserved Cardiac Function Heart Failure with an Aldosterone Antagonist (TOPCAT) and Aldosterone Receptor Blockade in Diastolic Heart Failure (ALDO-DHF)).^26^ The sodium-glucose cotransporter 2 inhibitor (SGLT2i) empagliflozin is also associated with a reduction in PICP at 52 weeks in HFpEF patients.^27^

Unlike SGLT2i and RAAS inhibitors, pirfenidone is a specific anti-fibrotic agent licensed for the treatment of idiopathic pulmonary fibrosis,^28^ with no haemodynamic effects.^29^ Preclinical data show that pirfenidone attenuates myocardial fibrosis through inhibition of TGF-ß, reduction in fibroblast proliferation and attenuation of myofibroblast differentiation.^1330^,^33^ The PIROUETTE trial was a phase 2 randomised controlled trial of pirfenidone versus placebo in patients with HFpEF and evidence of myocardial fibrosis (ECV≥27%).^14^ After 52 weeks of treatment, pirfenidone was associated with a reduction in ECV of 1.21%. Based on the results of observational studies, this magnitude of ECV reduction would be associated with a 9%–28% reduction in a composite of HF hospitalisation or all-cause mortality.^6^ 

After multivariable adjustment, there was an association between an increase in myocardial ECV and an increase in both PICP and CITP, suggesting altered collagen metabolism. No association was noted between baseline myocardial ECV and baseline biomarker concentrations. Although baseline concentrations of CITP were significantly increased in patients with ECV ≥27% compared with registry patients with ECV <27%, these results should be interpreted with caution as these patients differed from randomised patients with respect to baseline characteristics other than myocardial ECV (table 1). Moreover, ECV is an imperfect surrogate of fibrosis and may be affected by things other than collagen deposition, such as oedema. However, these results suggest that plasma collagen biomarkers may reflect dynamic changes in myocardial ECV to some extent. Pirfenidone treatment was associated with a transient reduction in CITP and increase in PICP:CITP ratio at 13 and 26 weeks, with no sustained effect observed at 52 weeks. This temporal pattern, which mirrors observations with RAAS inhibitors, suggests that these biomarkers may primarily reflect dynamic collagen turnover rather than collagen mass, although trial retention, treatment adherence, pharmacokinetic and pharmacodynamic tolerance may also be relevant.

This study has several limitations. All analyses were post hoc and performed on a small sample size, hence the study was underpowered and should be considered as hypothesis-generating. This study focused on measuring only two established collagen-derived peptides, PICP and CITP, but other promising circulating biomarkers of fibrosis, such as PIIINP, endotrophin^34^ and galectin-3,^35 36^ were not investigated. It is also possible that more potent anti-fibrotic interventions may have had a greater impact on circulating collagen biomarkers. Due to sample limitations, PICP and CITP were measured in plasma, while serum is the optimal matrix recommended by manufacturers. Larger, adequately powered studies are required to determine the relationship between plasma biomarkers of fibrosis and myocardial ECV and their response to specific anti-fibrotic therapy.

## Conclusions

In patients with HFpEF and more severe myocardial fibrosis (ECV≥27%), treatment with pirfenidone was associated with a trend towards an increase in log PICP:CITP ratio, with significant differences seen at 13 and 26 weeks, but not at 52 weeks. After multivariable adjustment, there was a significant direct association between changes in biomarker concentrations (log PICP and log CITP) and change in myocardial ECV. Blood biomarkers of collagen turnover and imaging to detect organ-specific fibrosis may have complementary roles for assessing the effects of anti-fibrotic therapies.

## Supplementary material

10.1136/openhrt-2025-003596 online supplemental file 1

## Data Availability

Data are available on reasonable request.
