# Supplementary material for: Effect of pirfenidone on plasma markers of collagen turnover in patients with heart failure, preserved left ventricular ejection fraction and myocardial fibrosis
Source: Open Heart. 2026 Feb 10;13(1):e003596. doi: 10.1136/openhrt-2025-003596 (PMC12911782; doi:10.1136/openhrt-2025-003596)
Supplement: online supplemental file 1 [file openhrt-13-1-s001.docx]

**The effect of pirfenidone on plasma markers of collagen turnover in patients with heart failure, preserved left ventricular ejection fraction and myocardial fibrosis**

**Supplemental file**

**Supplementary figure 1:** Distribution of (A) PICP, (B) CITP, and (C) PICP:CITP ratio at baseline and week 52.


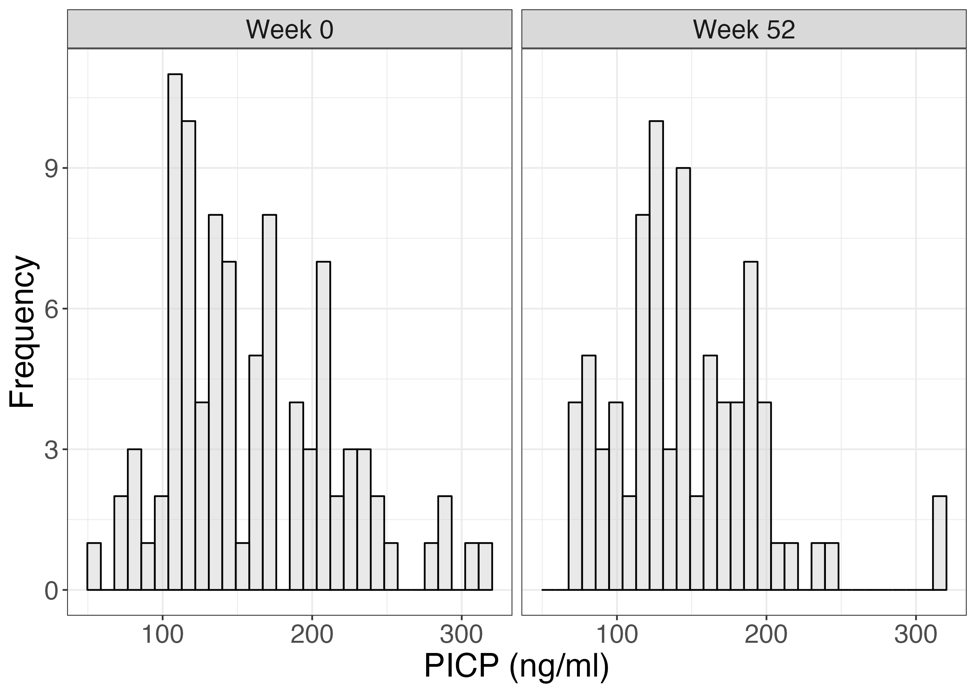


**A)**


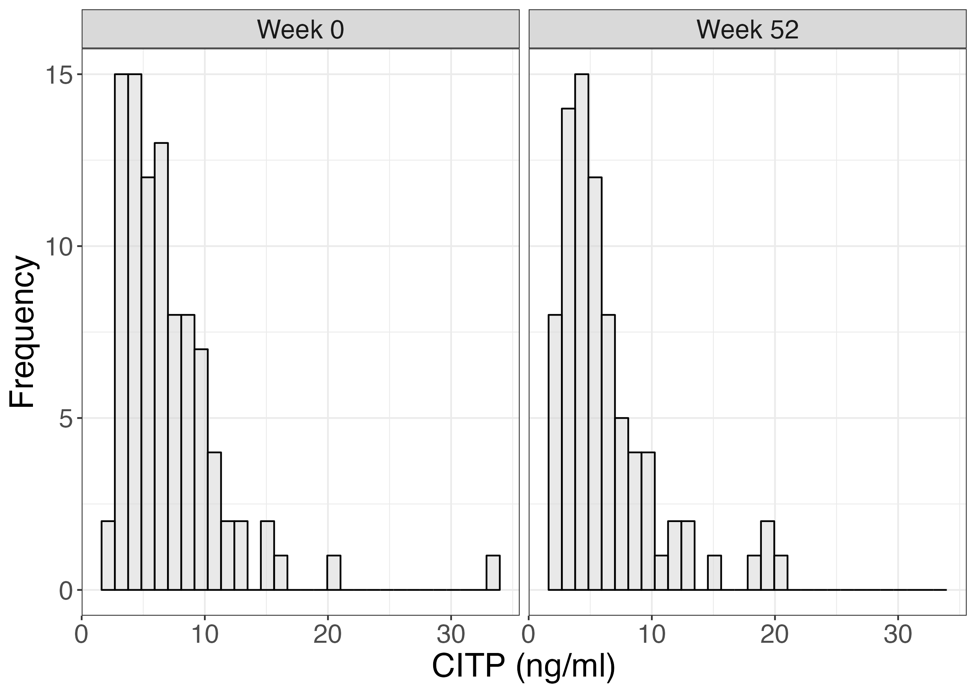


**B)**

**C)**


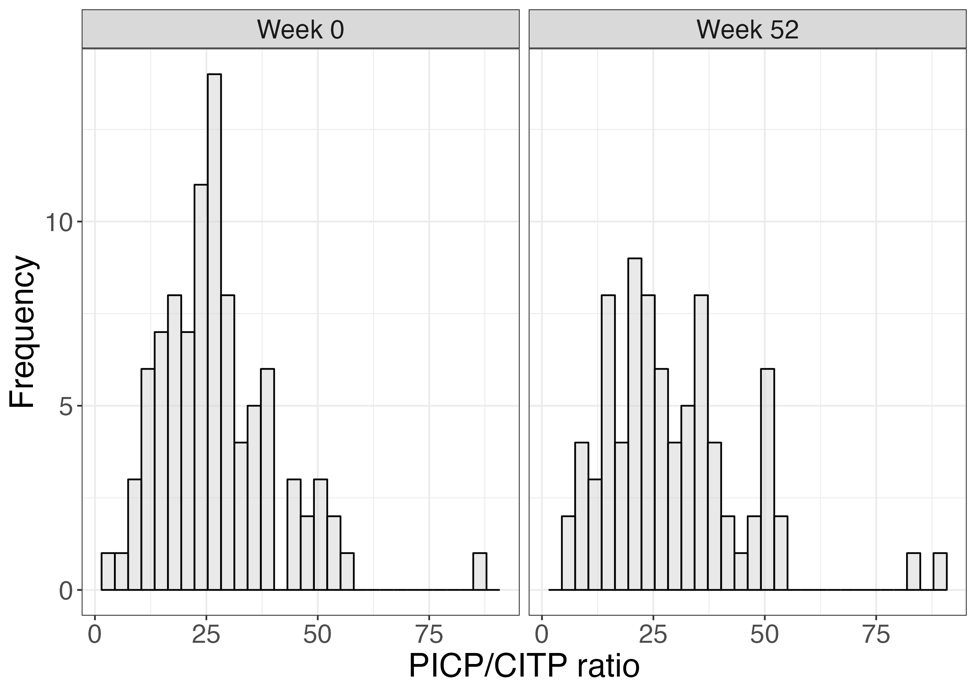


**Supplementary figure 2:** Distribution of log transformed (A) PICP, (B) CITP, and (C) PICP:CITP ratio at baseline and week 52.


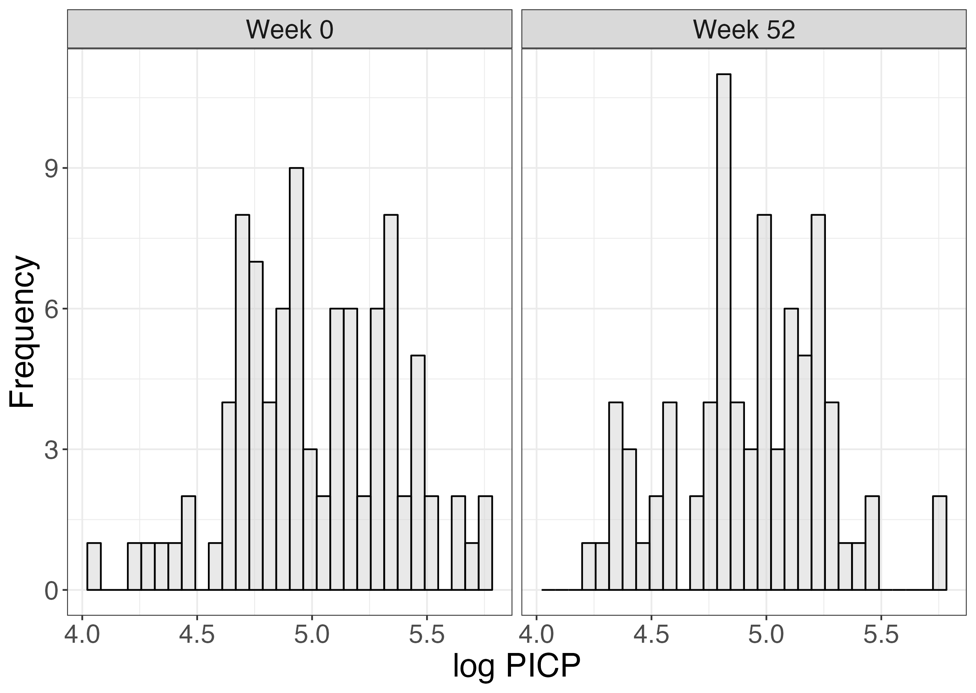


**A)**


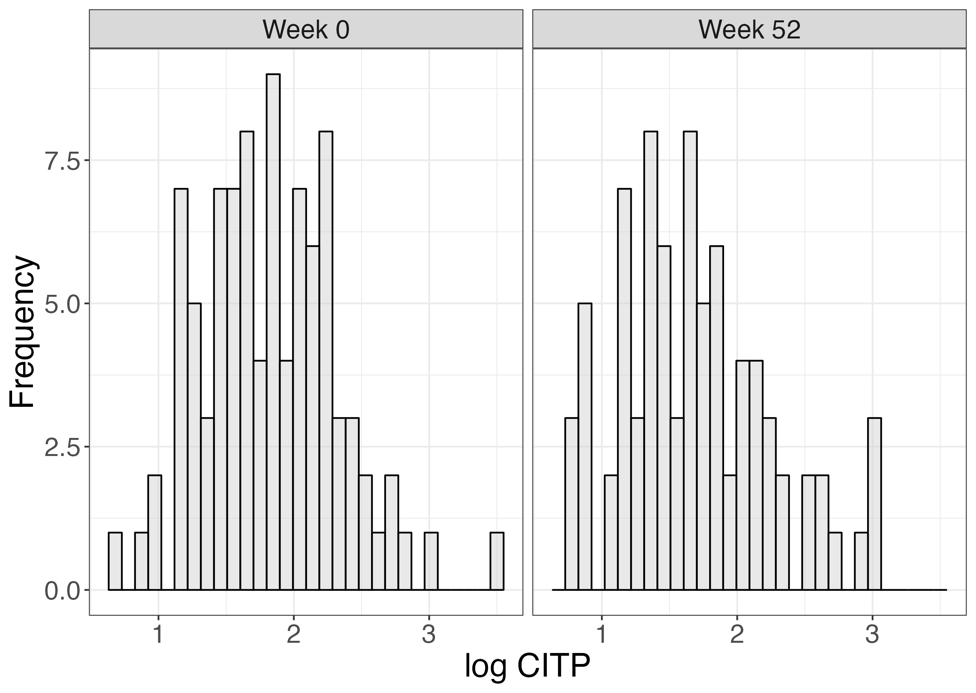


**B)**

**C)**


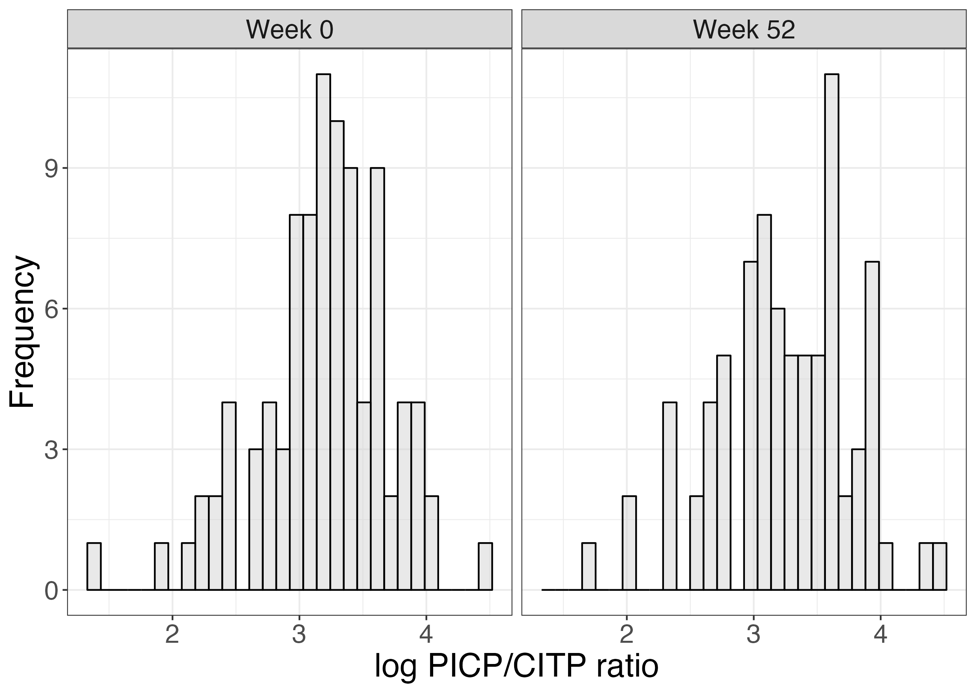


**Supplementary table 1**: Repeated measures mixed linear model for levels of log PICP, CITP and PICP:CITP ratio.

|  | **PICP** | | | | **CITP** | | | | **PICP:CITP ratio** | | | |
| --- | --- | --- | --- | --- | --- | --- | --- | --- | --- | --- | --- | --- |
|  | **Regression coefficient (SE)** | **95% CI** | **T statistic** | **P value** | **Regression coefficient (SE)** | **95% CI** | **T statistic** | **P value** | **Regression coefficient (SE)** | **95% CI** | **T statistic** | **P value** |
| **Intercept** | 1.545 (0.337) | 0.878 – 2.211 | 4.582 | <0.001 | 0.548 (0.180) | 0.192 – 0.903 | 3.048 | 0.003 | 0.921 (0.217) | 0.491 – 1.350 | 4.243 | <0.001 |
| **Baseline log level** | 0.667 (0.066) | 0.536 – 0.798 | 10.157 | <0.001 | 0.764 (0.064) | 0.636 – 0.891 | 11.949 | <0.001 | 0.615 (0.067) | 0.481 – 0.749 | 9.134 | <0.001 |
| **Female sex** | -0.043 (0.045) | -0.133 – 0.046 | -0.963 | 0.339 | -0.031 (0.058) | -0.146 – 0.084 | -0.538 | 0.592 | -0.006 (0.065) | -0.136 – 0.124 | -0.092 | 0.927 |
| **eGFR** | -0.000 (0.001) | -0.003 – 0.002 | -0.236 | 0.814 | -0.002 (0.002) | -0.006 – 0.001 | -1.185 | 0.240 | 0.003 (0.890) | -0.001 – 0.007 | 1.411 | 0.162 |
| **Pirfenidone** | 0.087 (0.069) | -0.051 – 0.225 | 1.260 | 0.211 | -0.142 (0.077) | -0.296 – 0.012 | -1.834 | 0.071 | 0.243 (0.089) | 0.065 – 0.420 | 2.725 | 0.008 |
| **Time (months)** | 0.006 (0.004) | -0.003 – 0.015 | 1.396 | 0.165 | -0.010 (0.007) | -0.023 – 0.004 | -1.454 | 0.148 | 0.016 (0.007) | 0.003 – 0.030 | 2.372 | 0.019 |
| **Interaction between pirfenidone and time** | -0.006 (0.006) | -0.019 – 0.007 | -0.939 | 0.350 | 0.012 (0.010) | -0.007 – 0.031 | 1.245 | 0.215 | -0.018 (0.010) | -0.038 – 0.001 | -1.871 | 0.064 |

CI = confidence interval, CITP = collagen type-1 C-terminal telopeptide, eGFR = estimated glomerular filtration rate, PICP = procollagen type-I C-terminal pro-peptide, SE = standard error

**Supplementary Table 2:** Univariable associations between change in myocardial ECV from baseline to week 52, change in log biomarkers, and baseline characteristics.

| **Variable** | **Univariable associations** | | | |
| --- | --- | --- | --- | --- |
|  | **Regression coefficient (SE)** | **95% CI** | **T statistic** | **P value** |
| Age (year)* | 0.11 (0.25) | -0.37 – 0.59 | 0.46 | 0.65 |
| Aorta distensibility (10^-3^/mmHg)* | 0.04 (0.24) | -0.43 – 0.51 | 0.17 | 0.86 |
| AF | 0.86 (0.46) | -0.04 – 1.75 | 1.87 | 0.07 |
| BMI (kg/m^2^)* | 0.13 (0.24) | -0.35 – 0.60 | 0.52 | 0.60 |
| **Change in log CITP*** | **0.50 (0.23)** | **0.05 – 0.95** | **2.17** | **0.03** |
| COPD | 0.08 (0.70) | -1.30 – 1.45 | 0.11 | 0.91 |
| Diabetes | 0.67 (0.53) | -0.37 – 1.70 | 1.25 | 0.21 |
| Ex-smoker | 0.77 (0.49) | -0.18 – 1.73 | 1.58 | 0.12 |
| Female sex | -0.71 (0.46) | -1.60 – 0.19 | -1.55 | 0.13 |
| Change in log GDF-15* | 0.36 (0.23) | -0.09 – 0.81 | 1.57 | 0.12 |
| GFR (ml/min/1.73m^2^)* | -0.02 (0.23) | -0.48 – 0.43 | -0.10 | 0.92 |
| Haemoglobin (g/dL)* | 0.03 (0.24) | -0.45 – 0.51 | 0.13 | 0.89 |
| Hyperlipidaemia | 0.29 (0.54) | -0.78 – 1.36 | 0.53 | 0.60 |
| Hypertension | 0.13 (0.63) | -1.11 – 1.36 | 0.20 | 0.84 |
| Change in HsTnT (pg/ml)* | -0.18 (0.24) | -0.64 – 0.29 | -0.74 | 0.46 |
| Infarct LGE | 0.30 (0.59) | -0.86 – 1.46 | 0.51 | 0.61 |
| Ischaemic heart disease | -0.03 (0.48) | -0.98 – 0.91 | -0.07 | 0.95 |
| LAVi (ml/m^2^)* | 0.31 (0.22) | -0.12 – 0.75 | 1.41 | 0.16 |
| LVEDVi (ml/m^2^)* | -0.14 (0.22) | -0.57 – 0.29 | -0.64 | 0.53 |
| LVEF (%)* | -0.35 (0.23) | -0.81 – 0.10 | -1.51 | 0.13 |
| LVMassi (g)* | 0.47 (0.22) | 0.03 – 0.91 | 2.10 | 0.04 |
| Non-white ethnicity | -1.00 (0.95) | -2.87 – 0.86 | -1.06 | 0.29 |
| Change in log NT-proBNP* | 0.24 (0.23) | -0.22 – 0.69 | 1.02 | 0.31 |
| **Change in log PICP*** | **0.51 (0.23)** | **0.06 – 0.96** | **2.23** | **0.03** |
| **Change in log PICP:CITP ratio*** | **-0.18 (0.23)** | **-0.64 – 0.28** | **-0.77** | **0.44** |
| Prior HF hospitalisation | 0.81 (0.77) | -0.70 – 2.31 | 1.05 | 0.29 |
| RAVI (ml/m^2^)* | 0.05 (0.22) | -0.39 – 0.48 | 0.21 | 0.84 |
| RVEDVi (ml/m^2^)* | -0.06 (0.24) | -0.52 – 0.41 | -0.23 | 0.82 |
| RVEF (%)* | -0.30 (0.23) | -0.76 – 0.16 | -1.29 | 0.20 |
| Sodium (mmol/L)* | 0.10 (0.24) | -0.36 – 0.56 | 0.42 | 0.68 |
| Stroke | 0.40 (0.73) | -1.03 – 1.84 | 0.55 | 0.59 |
| White cell count (10^9^/L)* | 0.36 (0.23) | -0.09 – 0.81 | 1.56 | 0.12 |

*Regression coefficients standardised to 1 standard deviation change in continuous variables

AF = atrial fibrillation, BMI = body mass index, CI = confidence interval, CITP = collagen type-1 C-terminal telopeptide, COPD = chronic obstructive pulmonary disease, ECV = extracellular volume, GDF-15 = growth differentiation factor 15, GFR = glomerular filtration rate, HsTnT = high sensitivity troponin T, LGE = late gadolinium enhancement, LAVi = indexed left atrial volume, LVEDVi = indexed left ventricle end diastolic volume, LVEF = left ventricle ejection fraction, LVMassi = indexed left ventricle mass, NT-proBNP = N-terminal pro B-type natriuretic peptide, HF = heart failure, PICP = procollagen type-I C-terminal pro-peptide, RAVi = indexed right atrial volume, RVEDVi = indexed right ventricle end diastolic volume, RVEF = right ventricle ejection fraction, SE=standard error

**Supplementary Table 3:** Multivariable adjusted associations between change in myocardial ECV and change in log PICP from baseline to week 52.

| **Variable** | **Multivariable associations (Adjusted R^2^ 0.16)** | | | |
| --- | --- | --- | --- | --- |
|  | **Regression coefficient (SE)** | **95% CI** | **T statistic** | **P value** |
| Intercept | -0.59 (0.36) | -1.30 – 0.12 | -1.66 | 0.10 |
| **Change in log PICP*** | **0.57 (0.22)** | **0.14 – 1.00** | **2.63** | **0.01** |
| Atrial fibrillation | 0.70 (0.44) | -0.17 – 1.57 | 1.60 | 0.11 |
| Diabetes | 0.88 (0.50) | -0.12 – 1.89 | 1.75 | 0.08 |
| Left ventricular mass indexed (g/m^2^)* | 0.49 (0.21) | 0.07 – 0.92 | 2.30 | 0.02 |
| Non-white ethnicity | -1.42 (0.90) | -3.22 – 0.38 | -1.58 | 0.12 |
| White cell count (x10^9^/L)* | 0.32 (0.22) | -0.11 – 0.75 | 1.48 | 0.14 |

*Regression coefficients standardised to 1 standard deviation change in continuous variables

CI = confidence interval, CITP = collagen type-1 C-terminal telopeptide , ECV=extracellular volume, PICP= procollagen type-I C-terminal pro-peptide, NT-proBNP = N-terminal pro B-type natriuretic peptide, SE=standard error

**Supplementary Table 4:** Multivariable adjusted associations between change in myocardial ECV and change in CITP from baseline to week 52.

| **Variable** | **Multivariable associations (Adjusted R^2^ 0.12)** | | | |
| --- | --- | --- | --- | --- |
|  | **Regression coefficient (SE)** | **95% CI** | **T statistic** | **P value** |
| Intercept | -0.53 (0.34) | -1.20 – 0.14 | -1.58 | 0.12 |
| **Change in log CITP*** | **0.46 (0.22)** | **0.01 – 0.90** | **2.05** | **0.04** |
| Atrial fibrillation | 0.82 (0.44) | -0.06 – 1.71 | 1.86 | 0.07 |
| Left ventricular mass indexed (g/m^2^)* | 0.42 (0.22) | -0.02 – 0.86 | 1.88 | 0.06 |
| White cell count (10^9^/L)* | 0.32 (0.22) | -0.12 – 0.76 | 1.47 | 0.15 |

*Regression coefficients standardised to 1 standard deviation change in continuous variables

CI = confidence interval, CITP = collagen type-1 C-terminal telopeptide , ECV=extracellular volume, PICP= procollagen type-I C-terminal pro-peptide, NT-proBNP = N-terminal pro B-type natriuretic peptide, SE=standard error

**Supplementary Table 5:** Multivariable adjusted associations between change in myocardial ECV and change in PICP:CITP ratio from baseline to week 52.

| **Variable** | **Multivariable associations (Adjusted R^2^ 0.07)** | | | |
| --- | --- | --- | --- | --- |
|  | **Regression coefficient (SE)** | **95% CI** | **T statistic** | **P value** |
| Intercept | -0.55 (0.34) | -1.23 – 0.14 | -1.59 | 0.12 |
| **Change in log PICP:CITP ratio*** | **-0.13 (0.23)** | **-0.59 – 0.32** | **-0.58** | **0.56** |
| Atrial fibrillation | 0.85 (0.46) | -0.06 – 1.76 | 1.86 | 0.07 |
| Left ventricular mass indexed (g/m^2^)* | 0.46 (0.23) | 0.01 – 0.91 | 2.04 | 0.05 |
| White cell count (10^9^/L)* | 0.30 (0.23) | -0.15 – 0.75 | 1.32 | 0.19 |

*Regression coefficients standardised to 1 standard deviation change in continuous variables

CI = confidence interval, CITP = collagen type-1 C-terminal telopeptide , ECV=extracellular volume, PICP= procollagen type-I C-terminal pro-peptide, NT-proBNP = N-terminal pro B-type natriuretic peptide, SE=standard error

**Supplementary Table 6:** Univariable associations between baseline myocardial ECV and baseline characteristics.

| **Variable** | **Univariable associations** | | | |
| --- | --- | --- | --- | --- |
|  | **Regression coefficient (SE)** | **95% CI** | **T statistic** | **P value** |
| Age (year)* | 0.00 (0.29) | -0.56 – 0.56 | -0.01 | 0.99 |
| Aorta distensibility (10^-3^/mmHg)* | 0.03 (0.29) | -0.53 – 0.59 | 0.09 | 0.92 |
| AF | 0.95 (0.57) | -0.16 – 2.06 | 1.68 | 0.10 |
| BMI (kg/m^2^)* | -0.76 (0.27) | -1.30 – 0.23 | -2.78 | 0.01 |
| **log CITP*** | **0.66 (0.28)** | **0.11 – 1.21** | **2.34** | **0.02** |
| COPD | 0.45 (0.85) | -1.22 – 2.12 | 0.53 | 0.60 |
| Current smoker | -1.20 (2.77) | -6.63 – 4.23 | -0.43 | 0.67 |
| Diabetes | -0.40 (0.62) | -1.62 – 0.81 | -0.65 | 0.52 |
| Ex-smoker | -0.08 (0.60) | -1.26 – 1.10 | -0.13 | 0.89 |
| Female sex | -0.66 (0.57) | -1.78 – 0.45 | -1.17 | 0.24 |
| log GDF-15* | 0.48 (0.28) | -0.07 – 1.04 | 1.72 | 0.09 |
| GFR (ml/min/1.73m^2^)* | -0.06 (0.29) | -0.62 – 0.50 | -0.21 | 0.84 |
| Haemoglobin (g/dL)* | -0.56 (0.28) | -1.10 – -0.01 | -1.98 | 0.05 |
| Hyperlipidaemia | -0.94 (0.66) | -2.24 – 0.36 | -1.41 | 0.16 |
| Hypertension | 0.12 (0.78) | -1.40 – 1.65 | 0.16 | 0.87 |
| HsTnT (pg/ml)* | 0.66 (0.28) | 0.12 – 1.21 | 2.39 | 0.02 |
| Infarct LGE | -0.02 (0.70) | -1.39 – 1.34 | -0.03 | 0.97 |
| Ischaemic heart disease | 1.04 (0.58) | -0.09 – 2.17 | 1.81 | 0.07 |
| LAVi (ml/m^2^)* | 0.70 (0.28) | 0.16 – 1.24 | 2.53 | 0.01 |
| LVEDVi (ml/m^2^)* | 0.01 (0.29) | -0.55 – 0.57 | 0.04 | 0.97 |
| LVEF (%)* | 0.17 (0.29) | -0.39 – 0.73 | 0.60 | 0.55 |
| LVMassi (g)* | 0.52 (0.28) | -0.03 – 1.07 | 1.84 | 0.07 |
| Non-white ethnicity | 0.57 (1.16) | -1.71 – 2.85 | 0.49 | 0.63 |
| log NT-proBNP* | 0.98 (0.27) | 0.46 – 1.51 | 3.69 | <0.001 |
| **log PICP*** | **0.36 (0.29)** | **-0.20 – 0.92** | **1.26** | **0.21** |
| **log PICP:CITP ratio*** | **-0.40 (0.29)** | **-0.96 – 0.16** | **-1.39** | **0.17** |
| Prior HF hospitalisation | -0.20 (0.78) | -1.72 – 1.33 | -0.25 | 0.80 |
| RAVI (ml/m^2^)* | 0.62 (0.28) | 0.07 – 1.17 | 2.23 | 0.03 |
| RVEDVi (ml/m^2^)* | 0.24 (0.28) | -0.31 – 0.80 | 0.86 | 0.39 |
| RVEF (%)* | 0.02 (0.29) | -0.54 – 0.58 | 0.08 | 0.93 |
| Sodium (mmol/L)* | -0.27 (0.28) | -0.83 – 0.28 | -0.96 | 0.34 |
| Stroke | 1.75 (0.90) | -0.02 – 3.52 | 1.94 | 0.06 |
| White cell count (10^9^/L)* | -0.36 (0.28) | -0.92 – 0.20 | -1.28 | 0.20 |

*Regression coefficients standardised to 1 standard deviation change in continuous variables

AF = atrial fibrillation, BMI = body mass index, CI = confidence interval, CITP = collagen type-1 C-terminal telopeptide, COPD = chronic obstructive pulmonary disease, ECV = extracellular volume, GDF-15 = growth differentiation factor 15, GFR = glomerular filtration rate, HsTnT = high sensitivity troponin T, LGE = late gadolinium enhancement, LAVi = indexed left atrial volume, LVEDVi = indexed left ventricle end diastolic volume, LVEF = left ventricle ejection fraction, LVMassi = indexed left ventricle mass, NT-proBNP = N-terminal pro B-type natriuretic peptide, HF = heart failure, PICP = procollagen type-I C-terminal pro-peptide, RAVi = indexed right atrial volume, RVEDVi = indexed right ventricle end diastolic volume, RVEF = right ventricle ejection fraction, SE=standard error

**Supplementary Table 7:** Multivariable adjusted associations between baseline myocardial ECV and baseline log PICP.

| **Variable** | **Multivariable associations (Adjusted R^2^ 0.29)** | | | |
| --- | --- | --- | --- | --- |
|  | **Regression coefficient (SE)** | **95% CI** | **T statistic** | **P value** |
| Intercept | 29.54 (0.44) | 28.66 – 30.42 | 66.84 | <0.001 |
| **log PICP*** | **0.33 (0.25)** | **-0.17 – 0.84** | **1.31** | **0.19** |
| AF | 1.00 (0.61) | -0.22 – 2.22 | 1.63 | 0.11 |
| BMI (kg/m^2^)* | -0.68 (0.26) | -1.20 - -0.16 | -2.60 | 0.01 |
| Haemoglobin (g/dL)* | -0.83 (0.25) | -1.33 - -0.33 | -3.31 | 0.001 |
| Hyperlipidaemia | -1.07 (0.59) | -2.25 – 0.11 | -1.81 | 0.07 |
| LVMassi (g)* | 0.47 (0.25) | -0.02 – 0.96 | 1.89 | 0.06 |
| log NT-proBNP* | 0.57 (0.30) | -0.02 – 1.16 | 1.92 | 0.06 |
| Stroke | 2.10 (0.82) | 0.46 – 3.74 | 2.55 | 0.01 |

*Regression coefficients standardised to 1 standard deviation change in continuous variables

AF=atrial fibrillation, BMI=body mass index, CI = confidence interval, CITP = collagen type-1 C-terminal telopeptide, ECV=extracellular volume, LVMassi=indexed left ventricular mass, PICP= procollagen type-I C-terminal pro-peptide, NT-proBNP = N-terminal pro B-type natriuretic peptide, SE=standard error

**Supplementary Table 8:** Multivariable adjusted associations between baseline myocardial ECV and baseline log CITP.

| **Variable** | **Multivariable associations (Adjusted R^2^ 0.30)** | | | |
| --- | --- | --- | --- | --- |
|  | **Regression coefficient (SE)** | **95% CI** | **T statistic** | **P value** |
| Intercept | 29.49 (0.44) | 28.61 – 30.36 | 66.99 | <0.001 |
| **log CITP*** | **0.41 (0.27)** | **-0.12 – 0.95** | **1.53** | **0.13** |
| AF | 1.09 (0.61) | -0.11 – 2.30 | 1.80 | 0.08 |
| BMI (kg/m^2^)* | -0.69 (0.26) | -1.20 - -0.17 | -2.63 | 0.01 |
| Haemoglobin (g/dL)* | -0.69 (0.26) | -1.21 - -0.17 | -2.68 | 0.01 |
| Hyperlipidaemia | -1.05 (0.59) | -2.23 – 0.13 | -1.78 | 0.08 |
| LVMassi (g)* | 0.57 (0.25) | 0.08 – 1.07 | 2.30 | 0.02 |
| log NT-proBNP* | 0.46 (0.31) | -0.15 – 1.06 | 1.49 | 0.14 |
| Stroke | 2.06 (0.82) | 0.43 – 3.68 | 2.51 | 0.01 |

*Regression coefficients standardised to 1 standard deviation change in continuous variables

AF=atrial fibrillation, BMI=body mass index, CI = confidence interval, CITP = collagen type-1 C-terminal telopeptide, ECV=extracellular volume, LVMassi=indexed left ventricular mass, PICP= procollagen type-I C-terminal pro-peptide, NT-proBNP = N-terminal pro B-type natriuretic peptide, SE=standard error

**Supplementary Table 9:** Multivariable adjusted associations between baseline myocardial ECV and baseline log PICP:CITP ratio.

| **Variable** | **Multivariable associations (Adjusted R^2^ 0.28)** | | | |
| --- | --- | --- | --- | --- |
|  | **Regression coefficient (SE)** | **95% CI** | **T statistic** | **P value** |
| Intercept | 29.49 (0.45) | 28.61 – 30.38 | 66.08 | <0.001 |
| **log PICP:CITP ratio*** | **-0.15 (0.27)** | **-0.69 – 0.40** | **-0.53** | **0.60** |
| AF | 1.13 (0.62) | -0.09 – 2.36 | 1.84 | 0.07 |
| BMI (kg/m^2^)* | -0.66 (0.26) | -1.19 - -0.14 | -2.51 | 0.01 |
| Haemoglobin (g/dL)* | -0.76 (0.27) | -1.29 - -0.23 | -2.84 | 0.01 |
| Hyperlipidaemia | -1.15 (0.60) | -2.33 – 0.04 | -1.93 | 0.06 |
| LVMassi (g)* | 0.55 (0.26) | 0.04 – 1.06 | 2.13 | 0.04 |
| log NT-proBNP* | 0.54 (0.31) | -0.07 – 1.15 | 1.76 | 0.08 |
| Stroke | 1.97 (0.83) | 0.32 – 3.61 | 2.38 | 0.02 |

*Regression coefficients standardised to 1 standard deviation change in continuous variables

AF=atrial fibrillation, BMI=body mass index, CI = confidence interval, CITP = collagen type-1 C-terminal telopeptide, ECV=extracellular volume, LVMassi=indexed left ventricular mass, PICP= procollagen type-I C-terminal pro-peptide, NT-proBNP = N-terminal pro B-type natriuretic peptide, SE=standard error

**Supplementary Table 10:** Levels of log PICP, CITP and PICP:CITP ratio in randomised vs registry patients. P value for two-sample t test.

|  | **Randomised (n-94)** | **Registry (n=13)** | **T statistic** | **P value** |
| --- | --- | --- | --- | --- |
| **log PICP** | 5.02 (0.4) | 4.92 (0.2) | 0.99 | 0.32 |
| **log CITP** | 1.82 (0.5) | 1.48 (0.4) | 2.33 | 0.02 |
| **log PICP:CITP ratio** | 3.19 (0.5) | 3.43 (0.4) | 1.62 | 0.11 |

Values are mean ± (standard deviation)

CITP = collagen type-1 C-terminal telopeptide, PICP = procollagen type-I C-terminal pro-peptide
